# Supplementary figures and images for: Impact of stabilizing mutations on the antigenic profile and glycosylation of membrane-expressed HIV-1 envelope glycoprotein
Source: PLoS Pathog. 2023 Aug 7;19(8):e1011452. doi: 10.1371/journal.ppat.1011452 (PMC10434953; doi:10.1371/journal.ppat.1011452)

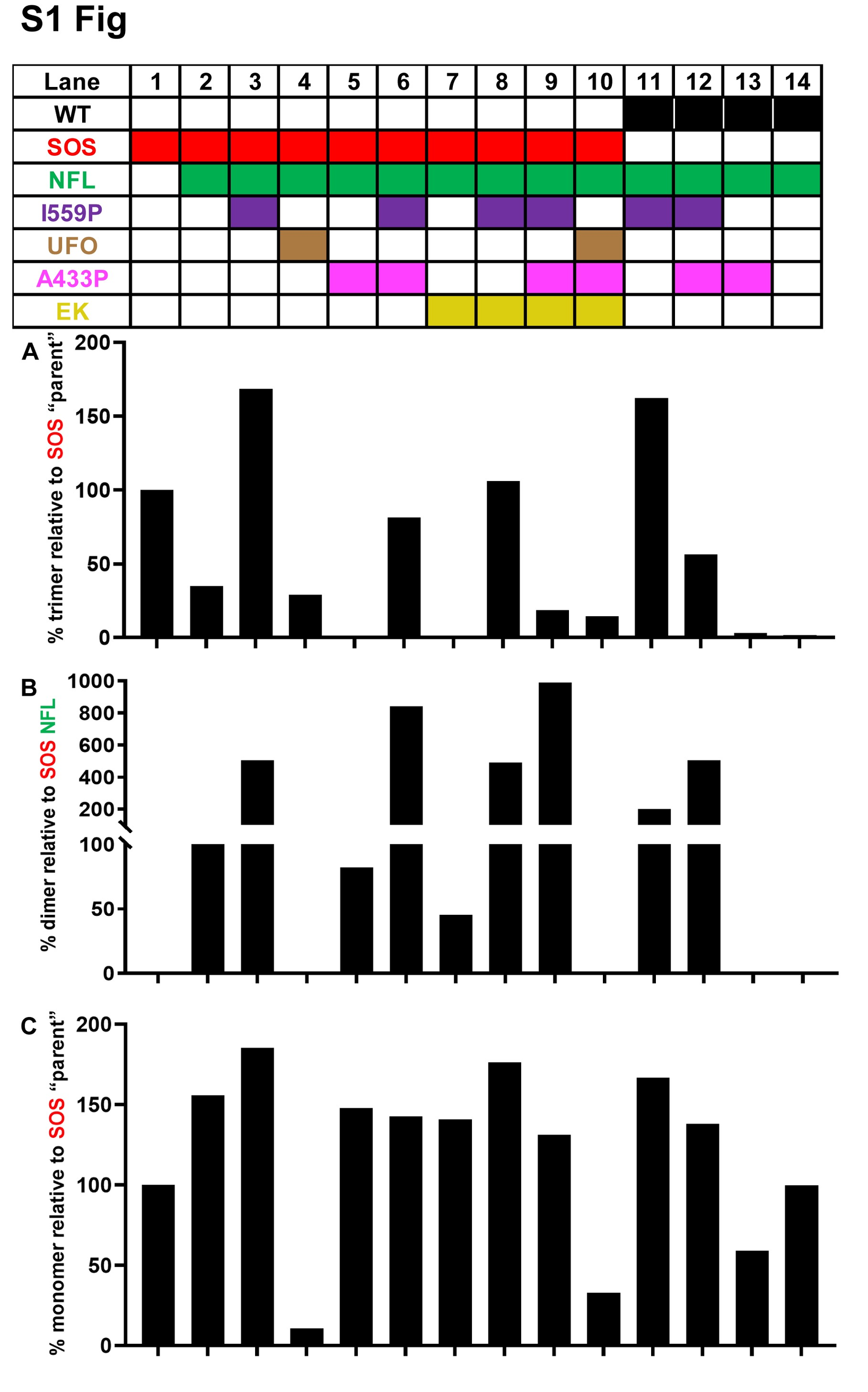

Supplement: S1 Fig — Related to Fig 2. Trimer and monomer bands were normalized against JR-FL gp160ΔCT SOS (Lane 1), while dimer bands were normalized against JR-FL gp160ΔCT SOS NFL (Lane 2). (TIF) [file ppat.1011452.s001.tif]

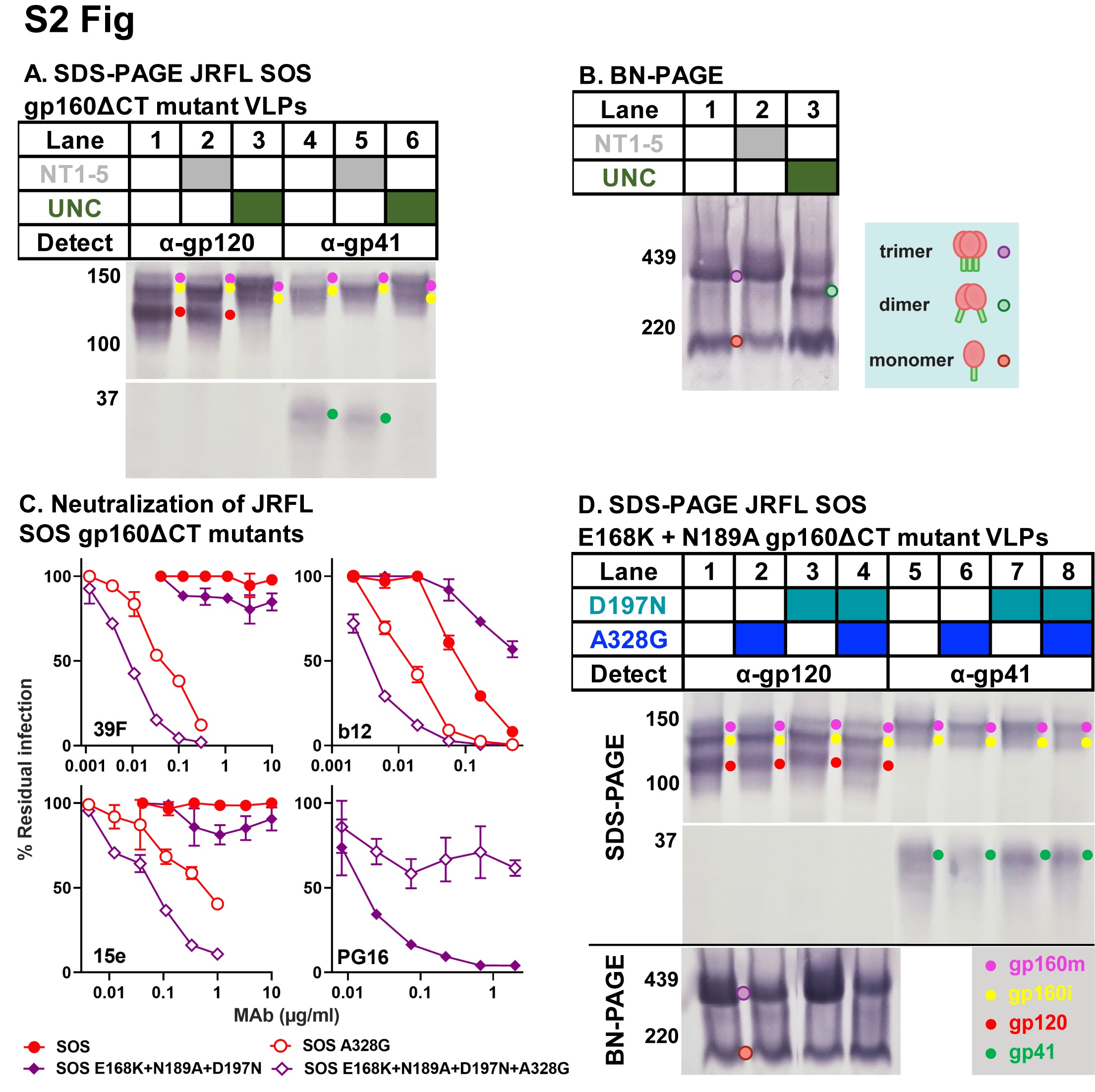

Supplement: S2 Fig — (A) JR-FL SOS parent, SOS NT1-5 (M535I+L543Q+N553S+Q567K+G588R) that also carries a D197N mutation, and SOS UNC (E168K+K510S+R511S) were analyzed by SDS-PAGE-Western blot, probed with anti-gp120 MAb (Lanes 1–3) or anti-gp41 MAb cocktail (Lanes 4–6). (B) The same samples from Part A were analyzed by BN-PAGE and probed with anti-gp120+gp41 MAb cocktail. (C) Neutralization sensitivity of JR-FL SOS gp160ΔCT pseudoviruses with A328G (open symbols) and without A328G (filled symbols) against b12, PG16, 39F, and 15e. Neutralization assay was performed in duplicates and repeated twice. Error bar represents the standard deviation of the mean. (D) Western blot analysis of the same mutants from Part (C) by SDS-PAGE probed with anti-gp120 MAb cocktail (Lanes 1–4) or anti-gp41 MAb cocktail (Lanes 5–8), and BN-PAGE probed with anti-gp120+gp41 MAb cocktail (bottom panel). Env species are indicated by colored dots. (TIF) [file ppat.1011452.s002.tif]

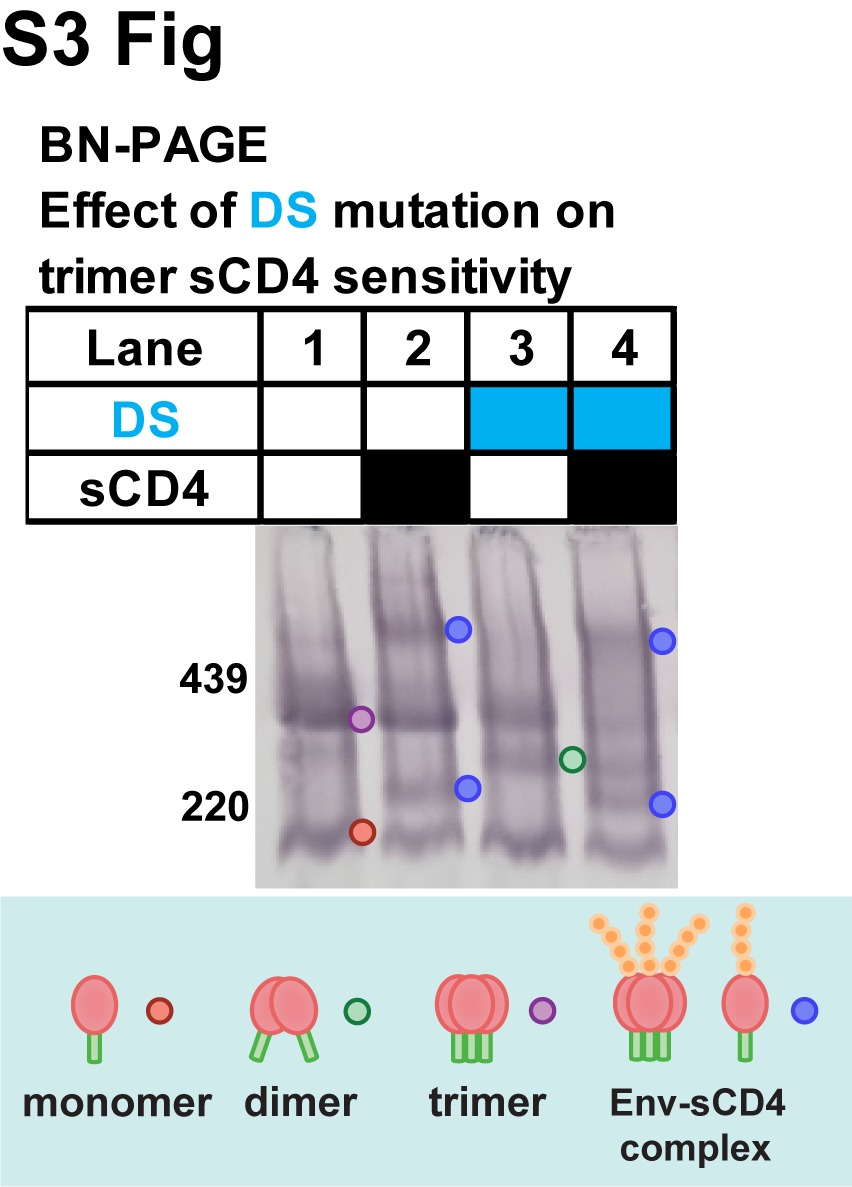

Supplement: S3 Fig — VLP mutants from lanes 15 and 18 of Figs 2 and 5 were mixed with 4-domain soluble CD4 or PBS, then washed, lysed, and analyzed by BN-PAGE-Western blot, probed with anti-gp120+gp41 MAb cocktail. Env species are indicated by colored dots. (TIF) [file ppat.1011452.s003.tif]

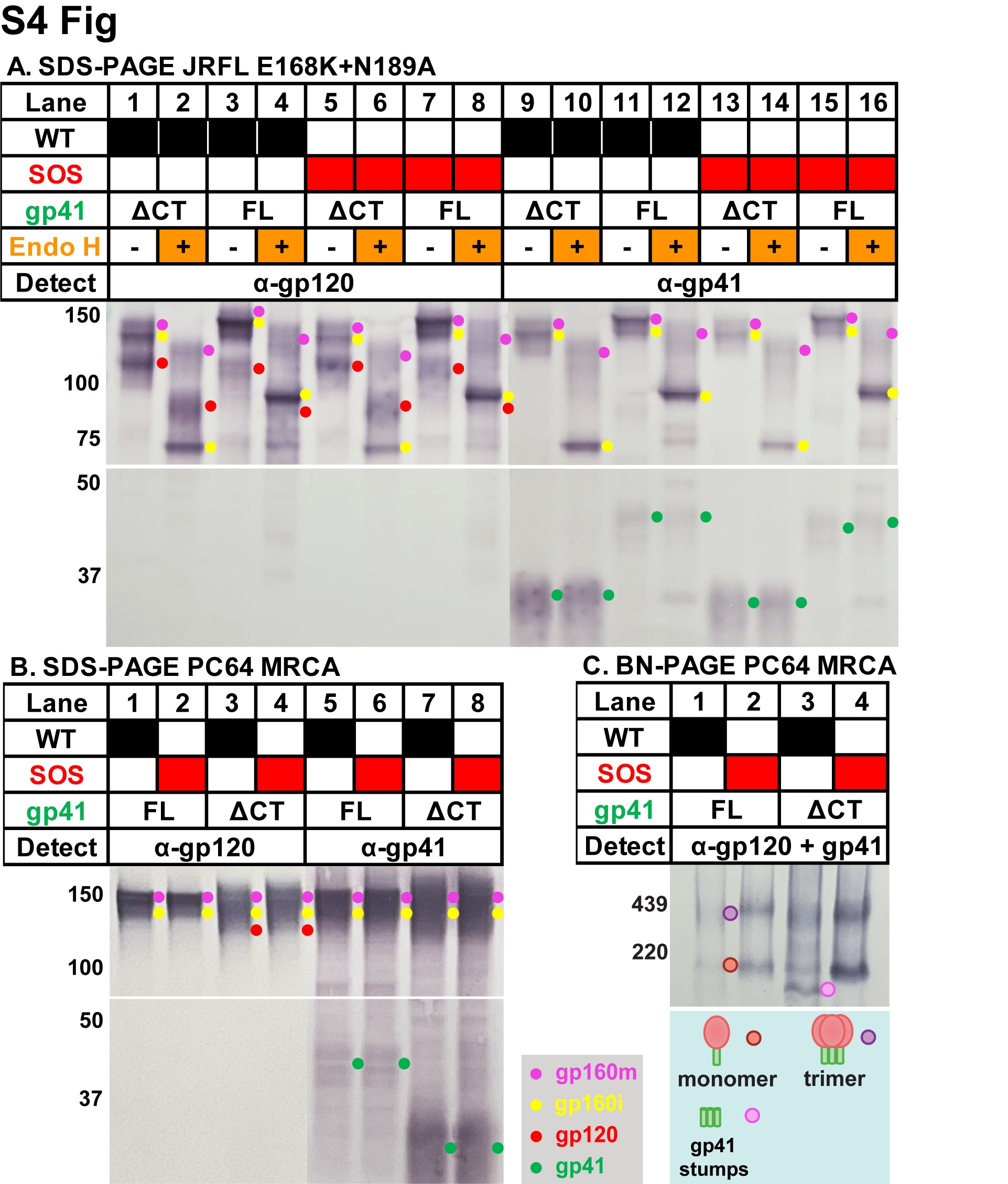

Supplement: S4 Fig — (A) JR-FL WT and SOS in gp160ΔCT and FL formats were denatured and digested with endo H and analyzed by SDS-PAGE-Western blot. Blot was probed with anti-gp120 MAb cocktail (Lanes 1–8) or anti-gp41 MAb cocktail (Lanes 9–16). (B) PC64 MRCA WT and SOS in gp160ΔCT and FL formats were analyzed by SDS-PAGE-Western blot, probed with anti-gp120 MAb cocktail (Lanes 1–4) or anti-gp41 MAb cocktail (Lanes 5–8). (C) The same mutants from Part (B) were analyzed by BN-PAGE Western blot probed with anti-gp120+gp41 MAb cocktail. Env species are indicated by colored dots. (TIF) [file ppat.1011452.s004.tif]

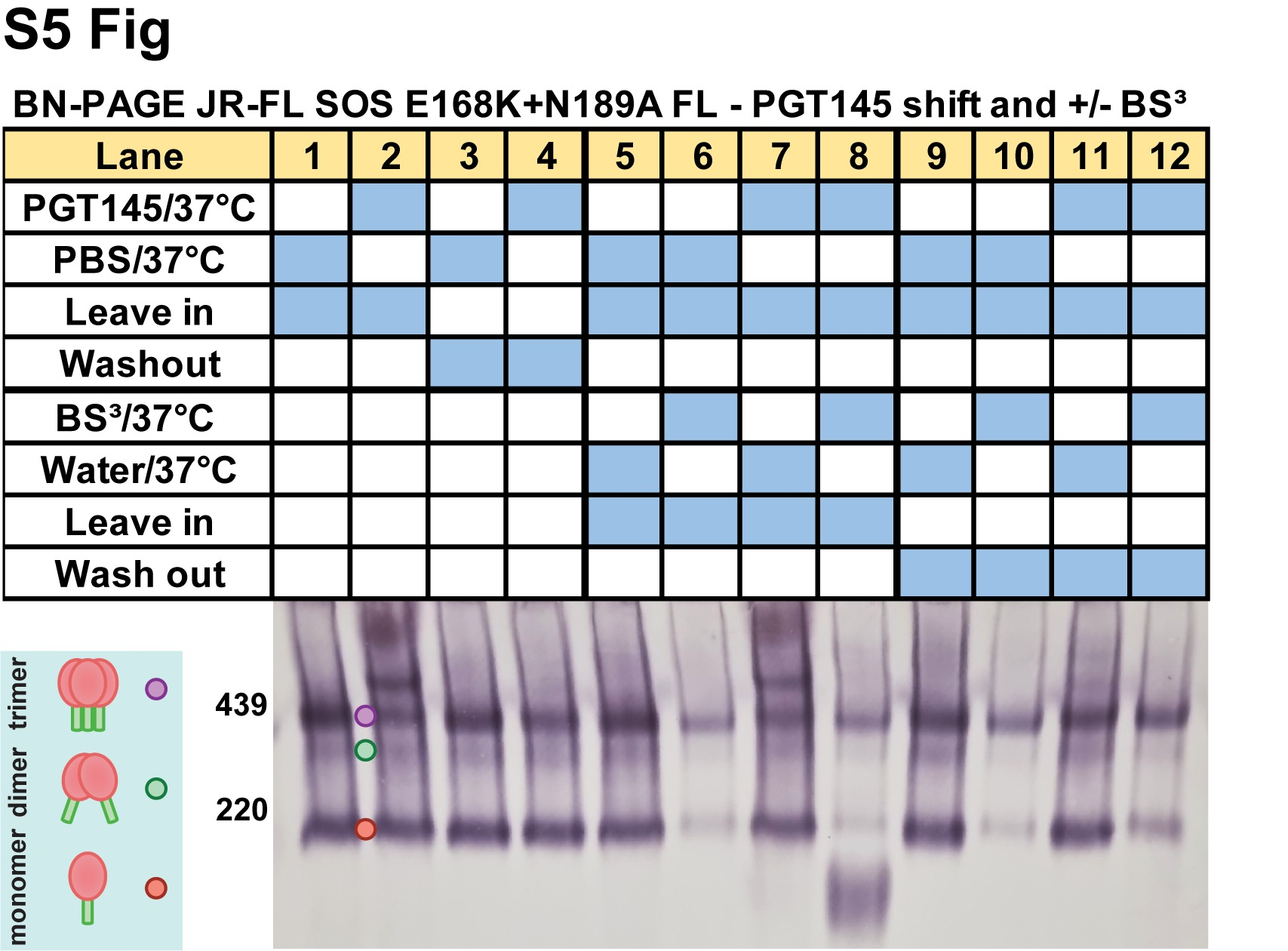

Supplement: S5 Fig — VLP bearing JR-FL SOS full length Env was incubated with PGT145 or PBS for 1h. The MAb-VLP complex was either wash with PBS (Wash out) or no washing (Leave in), followed by crosslinking with BS3. BS3 was either wash with PBS (Wash out) or no washing (Leave in), prior to electrophoresis on gel and probed with anti-gp120+gp41 MAb cocktail. (TIF) [file ppat.1011452.s005.tif]

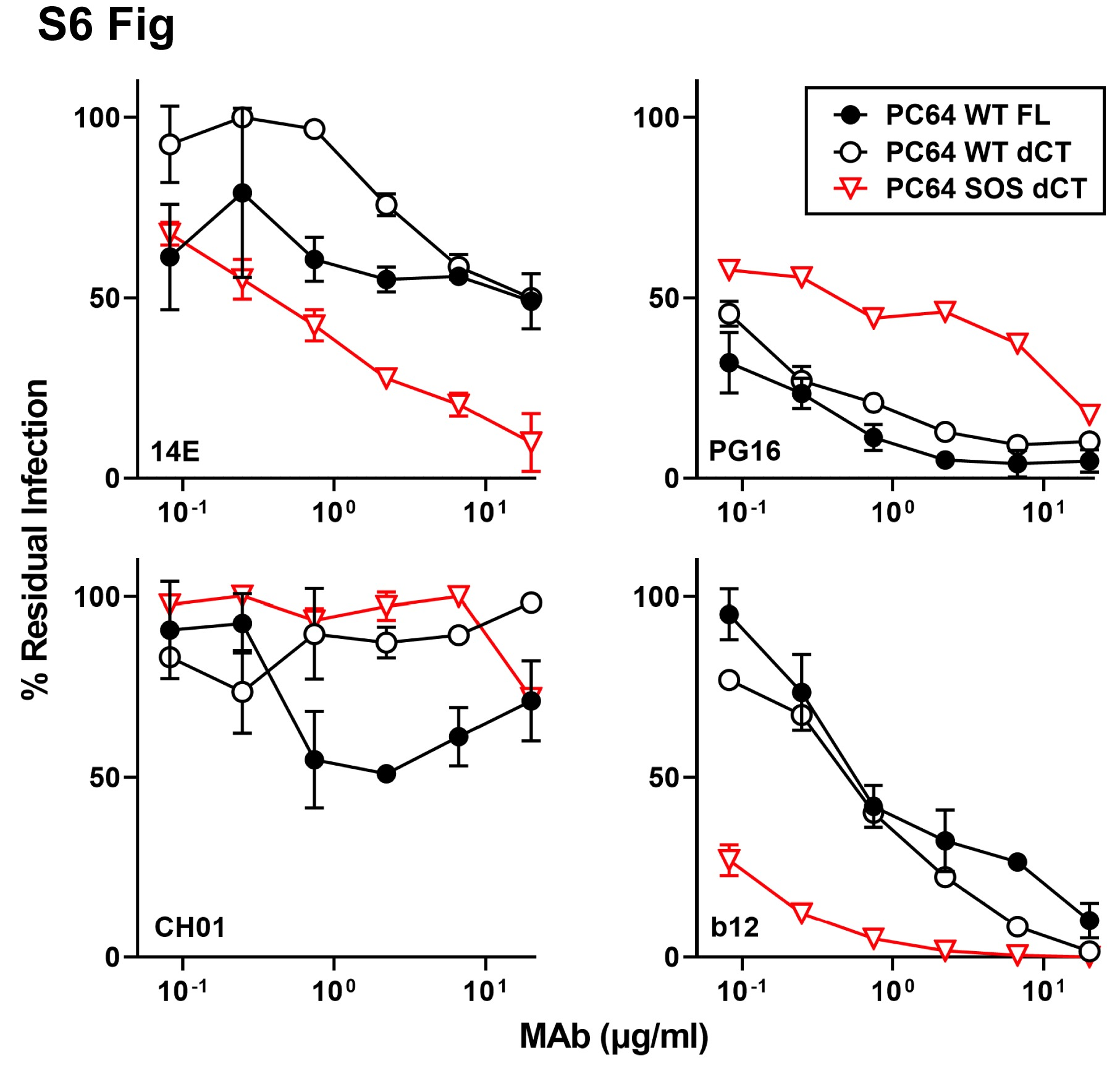

Supplement: S6 Fig — PC64 WT and SOS VLP bearing either gp160ΔCT or full-length (FL) were incubated with MAbs, and neutralization was measured using CF2Th.CD4.CCR5 as target cells. Filled symbols represent FL and open symbol represent gp160ΔCT. Neutralization assay was performed in duplicates and repeated twice. Error bar represents the standard deviation of the mean. (TIF) [file ppat.1011452.s006.tif]

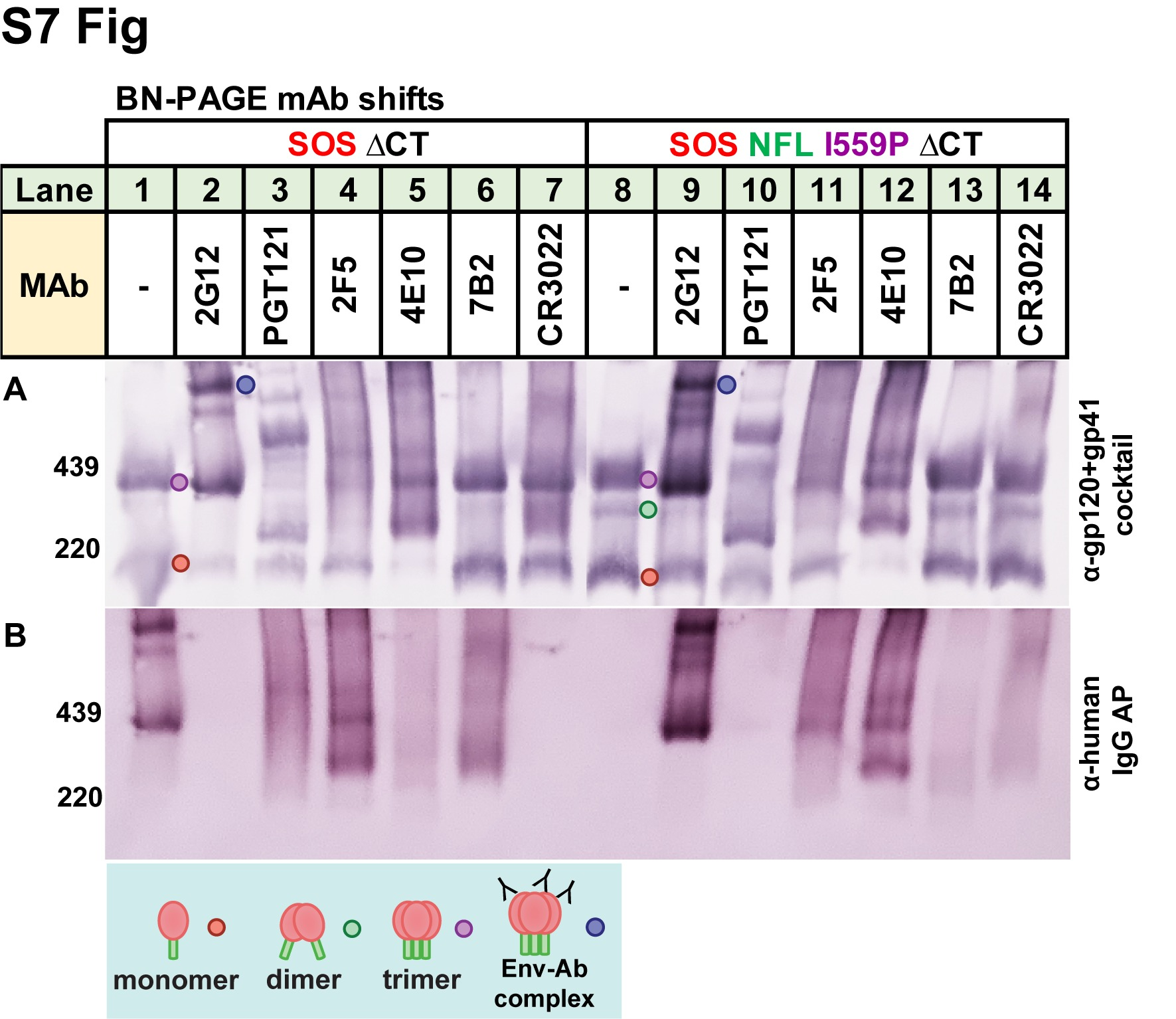

Supplement: S7 Fig — Related to Fig 7. JR-FL SOS and SOS NFL I559P gp160ΔCT mutants were analyzed in BN-PAGE shifts using selected MAbs. Duplicate blots were probed with (A) anti-gp120+gp41 MAb cocktail, followed by anti-human IgG AP conjugate, or (B) only the anti-human IgG AP conjugate. (TIF) [file ppat.1011452.s007.tif]

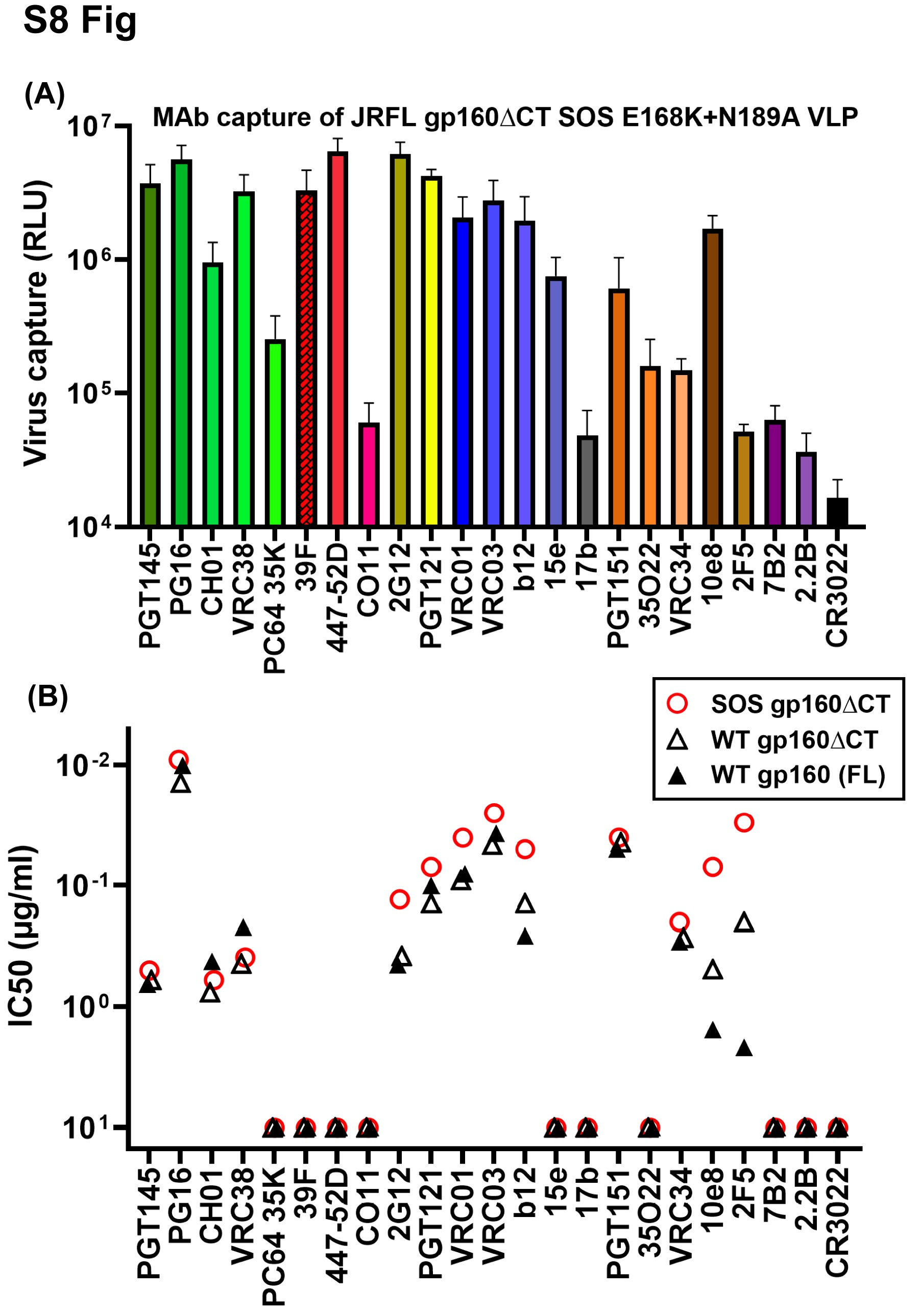

Supplement: S8 Fig — (A) Virus capture of JR-FL gp160ΔCT SOS E168K+N189A VLPs was evaluated using a large panel of MAbs. Values were presented as relative light unit (RLU). Virus capture assay was performed in quadruplicate and repeated at least two times. Error bars represent the standard deviation of the mean. (B) Neutralizing IC50s (μg/ml) of the same MAbs against SOS gp160ΔCT, WT FL and WT gp160ΔCT. JR-FL SOS FL was non-infectious and was not included in the data analysis. Neutralization assay was performed in duplicates and repeated twice. (TIF) [file ppat.1011452.s008.tif]

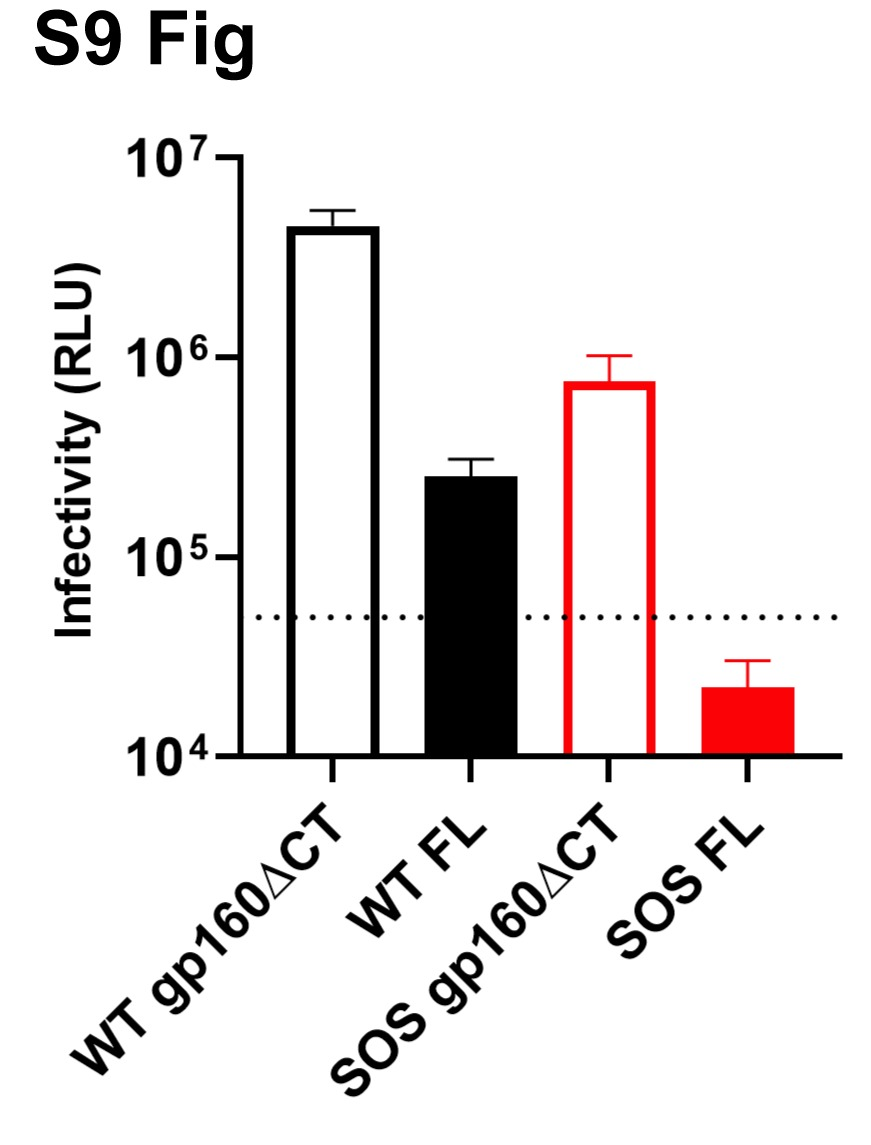

Supplement: S9 Fig — A cutoff at 50,000 RLU (relative light unit) was indicated as the minimum readout for productive infection. Filled bars represent FL and open bars represent gp160ΔCT. Infectivity was performed in triplicates and repeated at least twice. Error bar represents the standard deviation of the mean. (TIF) [file ppat.1011452.s009.tif]

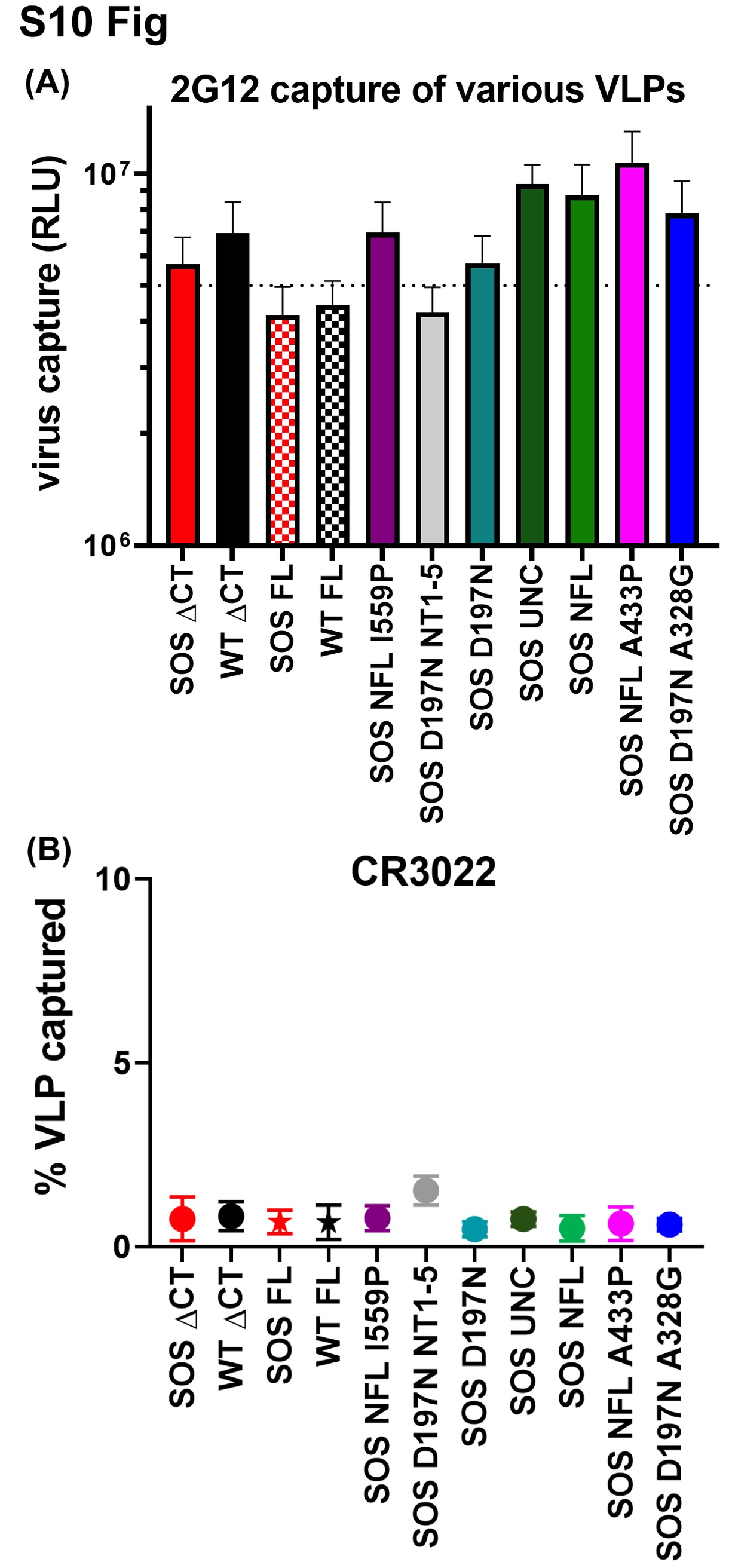

Supplement: S10 Fig — Virus capture assay was performed using (A) 2G12, or (B) CR3022 in quadruplicate and repeated at least three times. Error bars represent the standard deviation of the mean. (TIF) [file ppat.1011452.s010.tif]

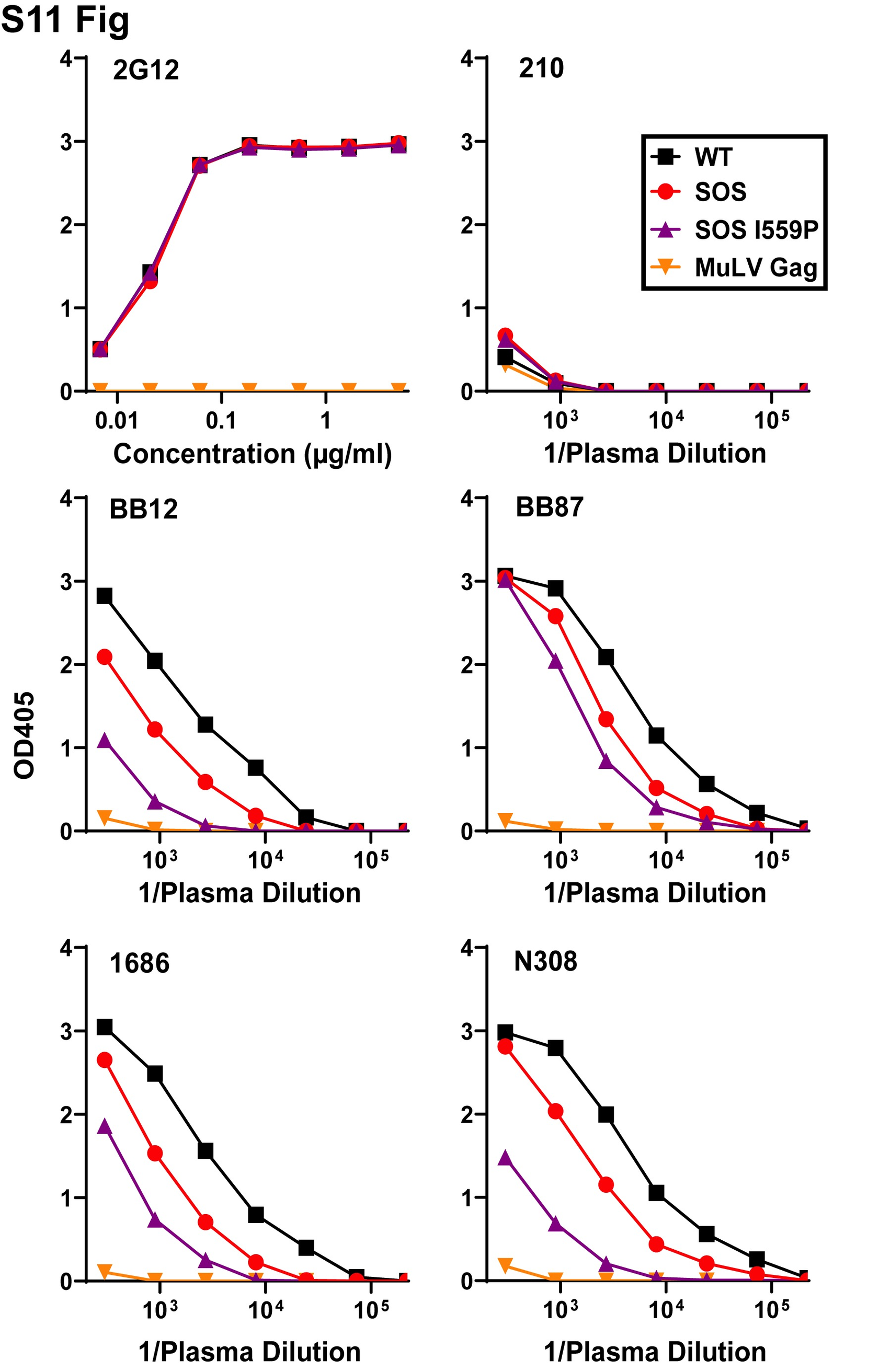

Supplement: S11 Fig — HIV+ plasma BB12, BB87, 1686 and N308, HIV- plasma 210, and 2G12 binding to JR-FL gp160ΔCT E168K+N189A WT, SOS or SOS I559P VLP was analyzed by ELISA. VLP ELISA was repeated twice and representative data is shown. (TIF) [file ppat.1011452.s011.tif]

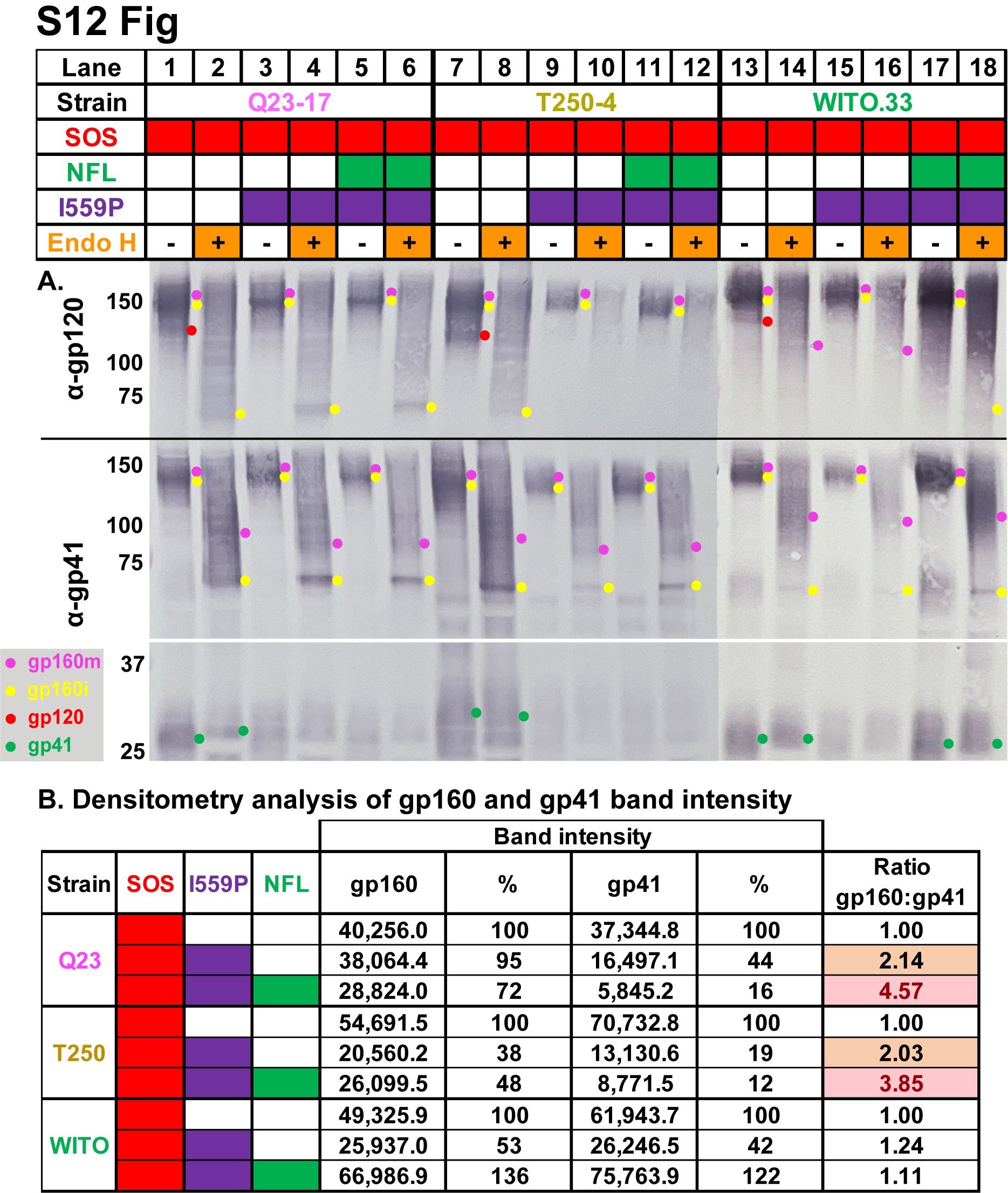

Supplement: S12 Fig — (A) VLPs expressing Q23-17 (Lanes 1–6), T250-4 (Lanes 7–12) and WITO.33 (Lanes 13–18), parent and mutant combinations were lysed and boiled in SDS/DTT, followed by digestion with endo H or PBS. Samples were analyzed in duplicate SDS-PAGE-Western blots probed with anti-gp120 MAb cocktail (top panel) or anti-gp41 MAb cocktail (bottom panels). Env species are indicated by colored dots. (B) Densitometry analysis of gp160 and gp41 band intensity for Part (A) to determine gp160:gp41 ratio. (TIF) [file ppat.1011452.s012.tif]

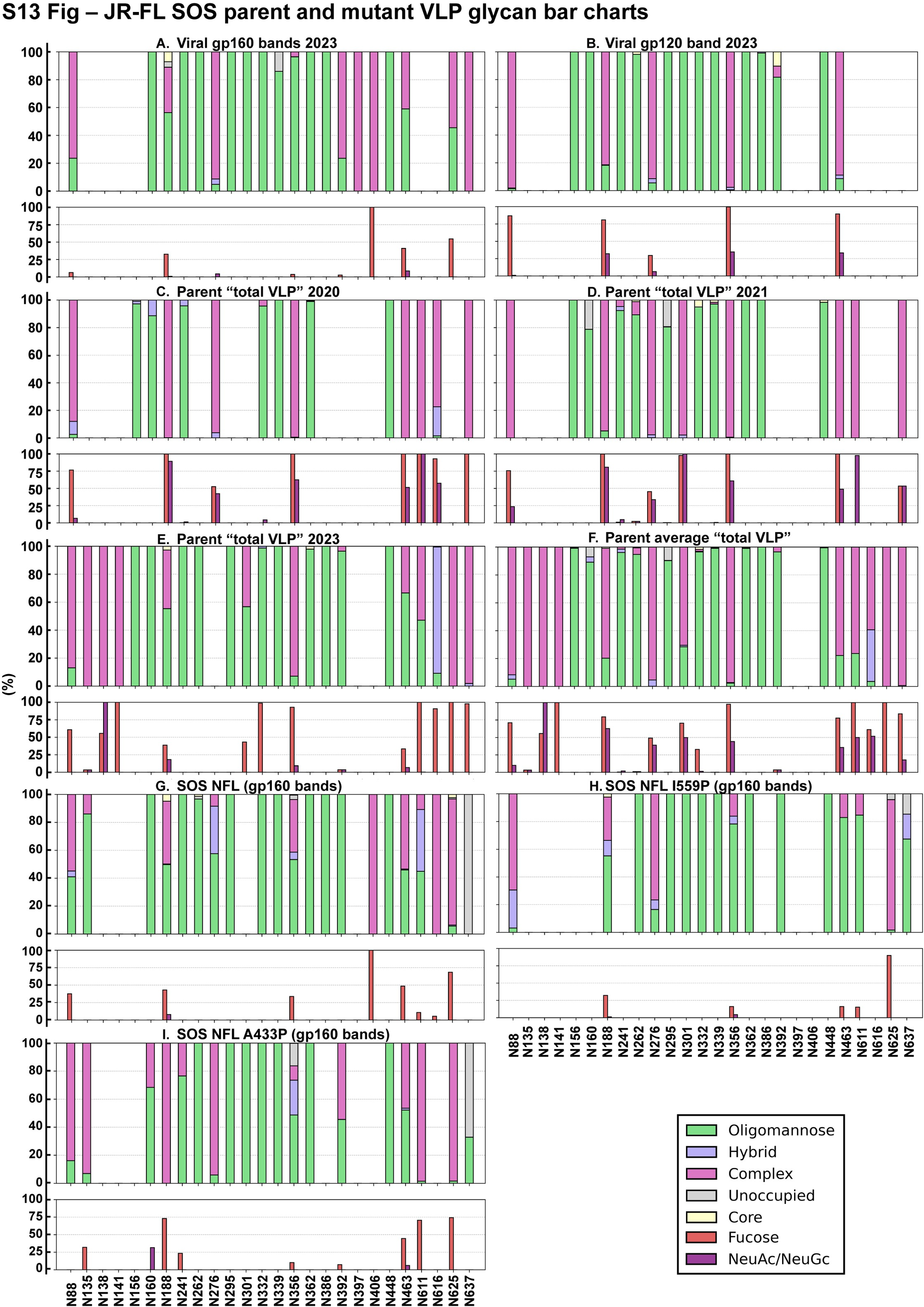

Supplement: S13 Fig — Related to S1 Main glycan analysis and S1 File and Figs S14–S15 and 11. Glycan maturation data of (A) viral gp160 and (B) viral gp120 derived from “parent” JR-FL gp160ΔCT SOS E168K+N189A VLP; parent “total VLPs” of JR-FL gp160ΔCT SOS E168K+N189A VLP preparation from (C) 2020, (D) 2021 and (E) 2023; (F) parent average of “total VLPs” of 2020, 2021 and 2023; gp160 bands derived from JR-FL mutant VLPs, namely (G) JR-FL SOS NFL, (H) SOS NFL I559P and (I) SOS NFL A433P. Glycan positions are numbered according to HxB2 strain. (TIF) [file ppat.1011452.s013.tif]

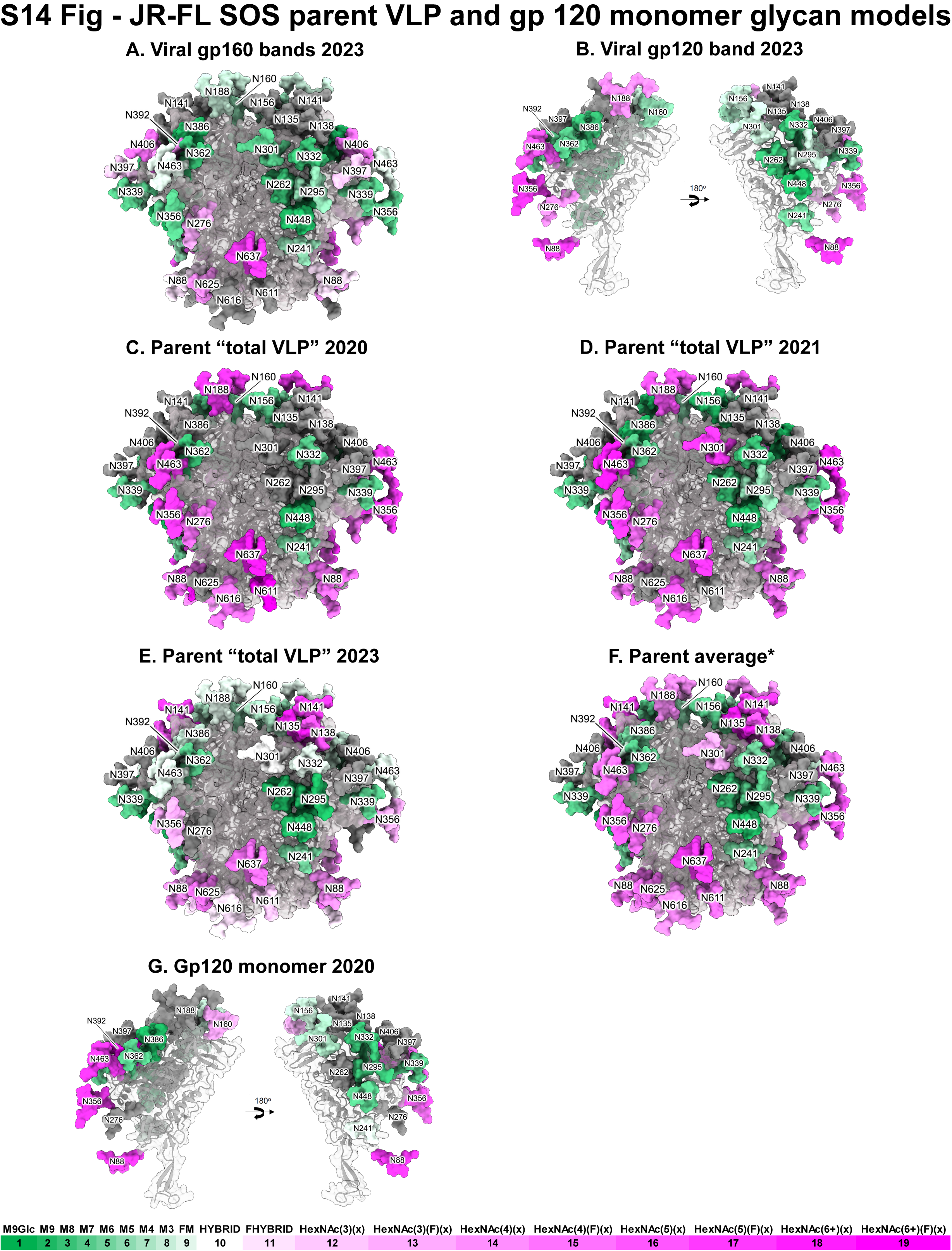

Supplement: S14 Fig — Related to Fig 11 and S1 Main glycan analysis. Models of JR-FL gp120 monomers and SOS E168K+N189A “parent” trimers (pdb: 6MYY) displaying colors that corresponds to the glycan scores. Glycan scores are colored in shades of green (high mannose) or magenta (complex). Untrimmed high mannose glycans are dark green while trimmed high mannose glycans are shown in lighter hues of green. Heavy complex glycans are shown in dark magenta, whereas smaller complex glycans are shown in lighter hues of magenta. Some glycans, rendered in gray, were not resolved in the JR-FL sample, and therefore have no score (not done, n.d.). These models were created from S1 Main glycan analysis. Models include (A) Viral gp160 derived from “parent” VLP 2023; (B) Viral gp120 derived from “parent” VLP 2023; (C-E) parent “total VLPs” samples from 2020, 2021 and 2023, respectively; (F) Glycan score average of parent “total VLPs” from 2020–2023; (G) gp120 monomer (2020) made by transfecting 293T cells and purified by GN-lectin. Glycan positions are numbered according to HxB2 strain. (TIF) [file ppat.1011452.s014.tif]

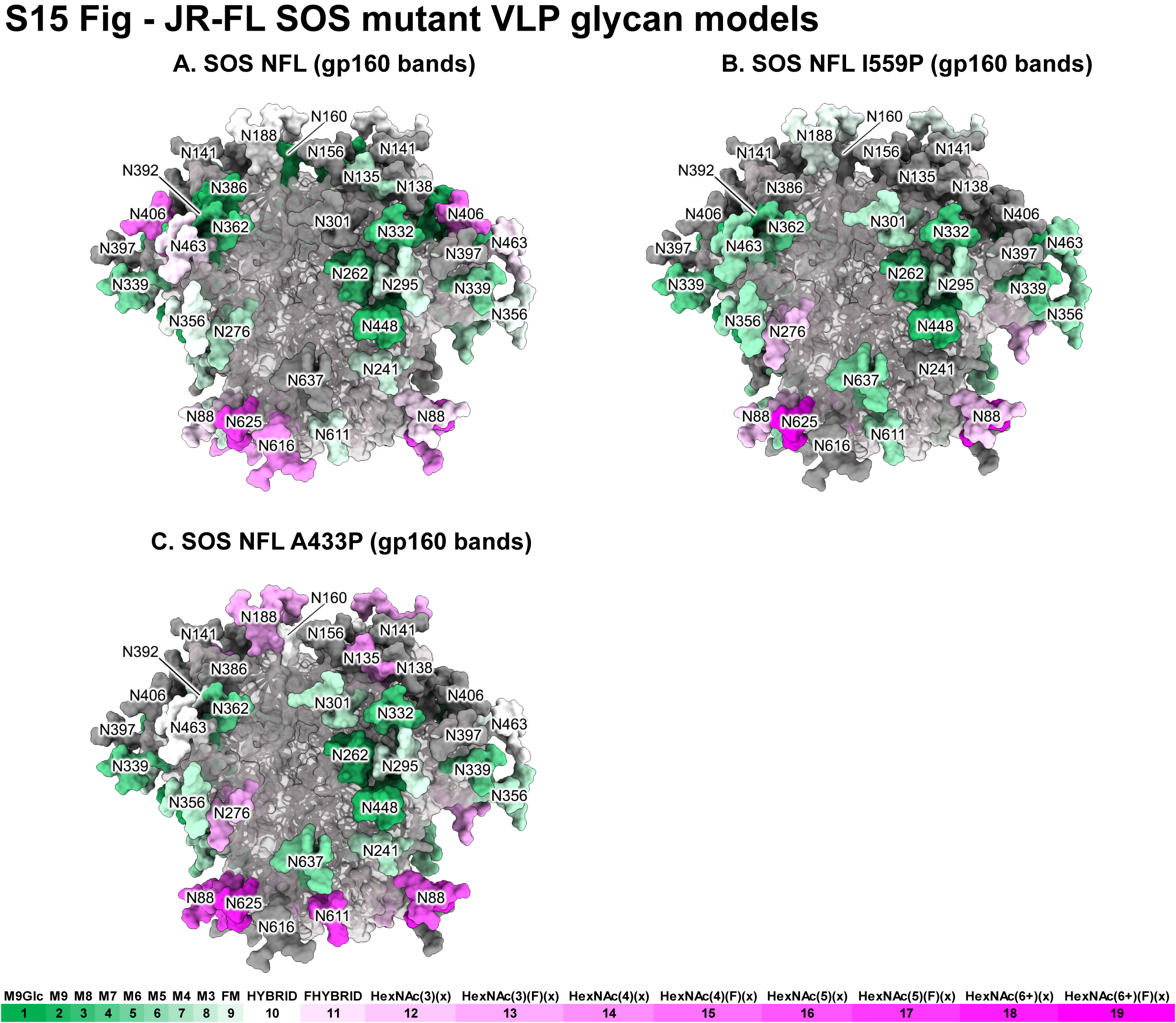

Supplement: S15 Fig — Related to Fig 11 and S1 Main glycan analysis. Models were created from data in S1 Main glycan analysis, whereby each model represents glycan profile of gp160 bands derived from (A) SOS NFL VLP; (B) SOS NFL I559P VLP; (C) SOS NFL A433P VLP. Glycan positions are numbered according to HxB2 strain. (TIF) [file ppat.1011452.s015.tif]
